# Mesopredator behavioral response to olfactory signals of an apex predator

**DOI:** 10.1007/s10164-016-0504-6

**Published:** 2017-01-10

**Authors:** Camilla Wikenros, Anders Jarnemo, Marielle Frisén, Dries P. J. Kuijper, Krzysztof Schmidt

**Affiliations:** 1 Grimsö Wildlife Research Station, Department of Ecology, Swedish University of Agricultural Sciences, 730 91 Riddarhyttan, Sweden; 2 Mammal Research Institute, Polish Academy of Sciences, Waszkiewicza 1, 17-230 Białowieża, Poland; 3 School of Business, Engineering and Science, Halmstad University, PO Box 823, 301 18 Halmstad, Sweden

**Keywords:** Interspecific interactions, *Lynx lynx*, Over-marking, Predator detection, *Vulpes vulpes*, Scent marking

## Abstract

Olfactory signals constitute an important mechanism in interspecific interactions, but little is known regarding their role in communication between predator species. We analyzed the behavioral responses of a mesopredator, the red fox (*Vulpes vulpes*), to an olfactory cue (scat) of an apex predator, the lynx (*Lynx lynx*) in Białowieża Primeval Forest, Poland, using video camera traps. Red fox visited sites with scats more often than expected and the duration of their visits was longer at scat sites than at control sites (no scat added). Vigilant behavior, sniffing and scent marking (including over-marking) occurred more often at scat sites compared to control sites, where foxes mainly passed by. Vigilance was most pronounced during the first days of the recordings. Red fox behavior was also influenced by foxes previously visiting scat sites. They sniffed and scent marked (multiple over-marking) more frequently when the lynx scat had been over-marked previously by red fox. Fox visits to lynx scats may be seen as a trade-off between obtaining information on a potential food source (prey killed by lynx) and the potential risk of predation by an apex predator.

## Introduction

Olfactory communication via scent marking is common among mammals (Eisenberg and Kleiman 1972; Brown and McDonald 1985). Marking substrates with urine, feces or secretions of scent glands is suggested to signal territory demarcation, resource possession, rank, and reproductive status, but may also help individual and group recognition (Ralls 1971; Johnson 1973; Gosling and Roberts 2001). However, as intraspecific signals can also attract predators (Cushing 1984; Sundell et al. 2003; Ylönen et al. 2003), there may be a trade-off between defending mates or resources and advertising presence to predators (Koivula and Korpimäki 2001; Rosell and Sanda 2006; Hughes et al. 2010). Predator scent can induce behavioral responses of prey, including decreased movements, increased vigilance, and relocation to safer sites (Lima 1998; Apfelbach et al. 2005; Zidar and Lövlie 2012; Kuijper et al. 2014; Wikenros et al. 2015). Whereas the costs and benefits of reciprocal interactions between predator and prey species (carnivores-herbivores) are straightforward, the role of scent marking in interspecific interactions between intraguild species remains largely unknown (Allen et al. 2016). Regarding carnivores, there is a potential for various types of interactions, ranging from competition and commensalism to predation (intraguild killing) (Palomares and Caro 1999). Studying the behavior of gray foxes (*Urocyon cinereoargenteus*) at puma (*Puma concolor*) scent marking sites, Allen et al. (2016) conclude that gray foxes use the puma scent to decrease predation risk, and also suggest that scent marking could be a mechanism that impacts species distribution and abundance.

Intraguild killing between two competing predator species has been documented for various taxa of mammalian carnivores and can affect the density and distribution of the subordinate species (Polis et al. 1989; Palomares and Caro 1999; Berger and Gese 2007). Intraguild killing is more likely to occur between species with high dietary overlap and large difference in body size (Donadio and Buskrik 2006). When facing the risk of predation, small-sized predators (mesopredators) may adjust their behavior to the presence of an apex predator. For instance, interspecific killing risk can drive smaller predators to trade off foraging for increased vigilance (Wikenros et al. 2014) or to avoid risky habitats (Fedriani et al. 1999, 2000).

The red fox (*Vulpes vulpes*, hereafter “fox”) and the Eurasian lynx (*Lynx lynx*, hereafter “lynx”) are competitors with a diet overlap consisting mainly of hares, rodents, birds, and roe deer (*Capreolus capreolus*) fawns (Jarnemo et al. 2004; Jarnemo and Liberg 2005; Odden et al. 2006; Panzacchi et al. 2008; Kidawa and Kowalczyk 2011; Krofel et al. 2011). However, fox and lynx focus on different main prey, with the smaller fox (4–10 kg) feeding on *Microtus* rodents and the larger lynx (15–25 kg) specializing in roe deer (Odden et al. 2006; Okarma et al. 1997). Besides their overlapping predation on live prey, fox scavenge deer carcasses that have been killed by lynx (Jędrzejewski et al. 1993; Jobin et al. 2000, Selva et al. 2005; Sidorovich et al. 2006), which leads to interspecific competition. Thus, besides indirect competition for food, lynx can also offer an important food source for foxes during periods of rodent scarcity (Helldin and Danielsson 2007). The potential for competitive interactions between lynx and fox may also be facilitated by their use of similar habitats (Kurki et al. 1998; Niedziałkowska et al. 2006).

In addition to the potential for competition and commensal relationships between an apex predator and a mesopredator, fox can be killed by lynx (Sunde et al. 1999; Helldin et al. 2006; Elmhagen et al. 2010). An exchange of interspecific olfactory signals may occur between lynx and fox, and reciprocal behavioral responsiveness to such signals can be anticipated. However, it is difficult to predict the behavioral response of fox to the olfactory signals of lynx, as they can either have a positive message (food availability) or a negative one (predation risk). Both lynx (Vogt et al. 2014) and fox (Macdonald 1979; Goszczyński 1990; Fawcett et al. 2013) use scent marks for intraspecific communication. It is also common for lynx to scent mark on top of marks from other individuals (Vogt et al. 2014). Such over-marking is common among terrestrial mammals and is indeed important in intraspecific communication (Ferkin and Pierce 2007). Observations of interspecific over-marking, however, are less common in the literature.

We studied behavioral responses of fox to olfactory cues (scats) of lynx in Białowieża Primeval Forest (hereafter “BPF”), Poland, with an experimental approach. Our aim was to determine whether the response of the fox to an olfactory cue of lynx was:

1. Neutral, i.e., similar visitation frequency and behavior at scat sites and control sites.
2. Suggested fear, either by avoidance or increased vigilance at scat sites.
3. Suggested attraction, by increased visitation frequency, sniffing, or scent marking (including over-marking lynx scat), at scat sites.

Due to the possibility of both negative and positive messages of lynx scats for the fox, we did not expect a higher frequency of any particular behavioral response by fox. However, we expected that fox confronted with the odor of the larger, potentially risky predator may show avoidance of sites with signs of lynx.

## Materials and methods

### Study area

The BPF is a temperate mixed lowland forest spanning 1450, of which 600 km^2^ lies in Poland (52°45′N, 23°50′E), where this study was conducted, and the rest in Belarus. BPF contains Białowieża National Park (105 km^2^), with 47 km^2^ of its area proclaimed a strict reserve. BPF consists of rich, multi-species tree stands with five main forest types occurring along gradients of soil richness and water availability (Faliński 1986; Bernadzki et al. 1998). This study was conducted in the part of the BPF managed for forestry purposes. The managed forest differs from the strictly protected stands in tree species composition, with more coniferous forest and a younger average age of the tree stands (Jędrzejewska et al. 1994). The mean annual air temperature in the area is 7 °C. The monthly mean temperature is lowest in January (−5 °C) and highest in July (18 °C). Average daily temperatures during the study period were 7 °C in October and 4 °C in November 2012. Mean precipitation is 641 mm/year and snow covers the ground for 144 days, on average, annually. Lynx has been a protected species in Poland since 1989; they occur in BPF at densities of around 1–3 lynx/100 km^2^ (Schmidt et al. 2009). Population densities of foxes averaged 20–30 foxes/100 km^2^ (Jędrzejewska and Jędrzejewski 1998).

### Field methods

We recorded the animals’ behavior during autumn months by video camera traps distributed in 54 sites designed for monitoring ungulate behavior in another study (Wikenros et al. 2015). The sites were situated along forest edges facing agricultural fields. We attached cameras to trees, and directed them at the agricultural fields at a height of approximately 1 m and at angles that ensured good visibility of fields on the recordings. We used lynx scats to simulate predator presence at 27 sites. Scats were collected from two captive mature female lynx (outside the mating season) that were fed on a diverse diet, including wild ungulate carcasses. Scats were kept frozen at −20 °C for a few days up to 1 month until the experiment started. Disposable gloves were used to prevent transmission of human smell to the scats. We randomly assigned locations as being a scat site or control site (no lynx scat added) with both treatment and control sites in all fields. The distance between scat sites and control sites averaged 115 m (±10 SE, range 50–270). Scats were placed in the centre of the detection area of the cameras, at a distance of approximately 10 m from the camera. Scat and control sites were recorded with movement- and body-heat-triggered passive sensor cameras (Digital Trail Camera SGN-5220) that automatically switched from color mode, during the day, to infrared mode (black and white videos) at night. This allowed the recording of behavioral responses for 24 h/day. Cameras were set to record for 60 s when triggered. Preliminary tests showed that the sensors had a detection range of 24–27 m over an area of approximately 100 ° and recorded all animals inside the reception area. The experiments started on 9, 12 and 24 October and 9 November 2012, and each site was monitored during 12 consecutive days. Due to occasional malfunctioning of the cameras, sites were recorded during 485 days in total (of which 251 days were at experimental sites) out of 648 days. We assumed that recordings that started within a 5-min interval were due to the same individual and pooled these recordings.

### Classification of behavior

We classified the behavior of fox using the following behavioral classes:

1. Passing by, when walking or trotting (no other observed behavior).
2. Vigilant, when standing still with the head erect, looking around.
3. Sniffing, when the head pointed to the ground, not foraging.
4. Scent marking, when urinating or defecating; this was defined as over-marking when it occurred on top of a lynx scat and as multiple over-marking when it occurred on top of a lynx scat that had been previously over-marked by fox.

We excluded one recording with more than one fox at a control site to avoid classification of behavior that may have been directed towards a conspecific. We also excluded three recordings that lasted less than 2 s from the analyses of duration of visit and behavior. Neither the scat sites nor the control sites were visited by lynx during the study period.

### Statistical analyses

We tested if the visitation frequency (number of visits) and duration of individual visits by foxes differed between scat and control sites using a χ^2^ goodness-of-fit test and *t*-test, respectively. We tested if the behavior of fox [separate models for passing by, vigilant, sniffing, and scent marking (including over-marking and multiple over-marking)] differed between scat sites and control sites using logistic regression with presence (1) or absence (0) of a given behavior as a response variable. We used site identity as a random effect to account for repeated measurements in all models. First, we used treatment (two-level categorical variable; scat or control) as an explanatory variable to test if the presence of lynx scat affected fox behavior. Secondly, we excluded control sites and used time since the experiment started (continuous variable; 0–12 days) as an explanatory variable to test if the freshness of the scat affected fox behavior. In addition, because over-marking of foxes that previously visited sites may influence the behavior of later visitors, we also included fox over-marking (two-level categorical variable; 0 or 1, where 1 was given for all behaviors after an over-marking by fox) as an explanatory variable. All analyses were conducted in R version 3.2.2 (R Development Core Team 2015) using the lme4 package (Bates et al. 2014). We considered *p* < 0.05 to be statistically significant.

## Results

We recorded 102 fox visits; 75 at 17 scat sites and 27 at 11 control sites. Fox visits lasted 2331 s (38 min) altogether, including 51 s on the control sites. Foxes visited scat sites more often (75 out of 102) than controls (27 out of 102) if expecting equal visitation frequency (*χ* ^2^ = 22.59, *df* = 1, *p* < 0.0001). The duration of visits was longer at scat sites (mean ± SE = 31 ± 4 s, *n* = 73) than at control sites (mean ± SE = 2 ± 0.4 s, *n* = 25, *t* = −7.446, *p* < 0.001).

**Fig. 1:**
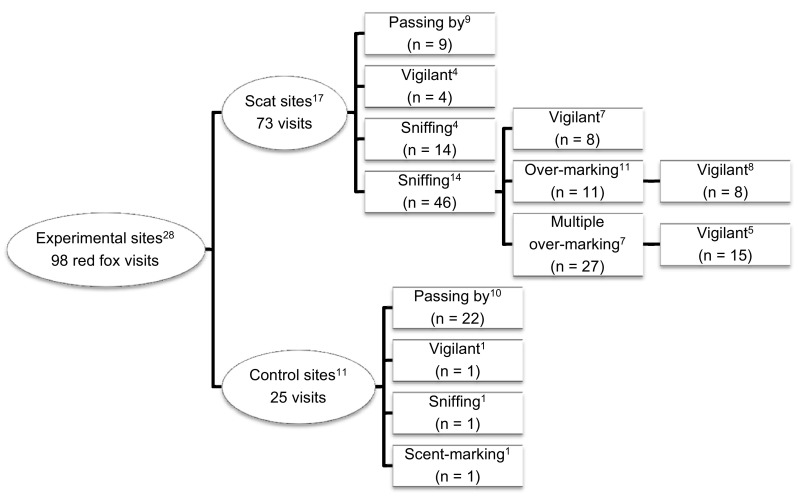
Recorded behavior of red foxes (different behavioral responses are illustrated within *boxes*) at experimental sites with lynx scats or control sites without scats. The number of sites where the different behaviors were expressed is shown by *superscript letters*. The observations were recorded by movement- and body-heat-triggered passive sensor cameras in the Białowieża Primeval Forest, Poland, during autumn 2012

**Fig. 2:**
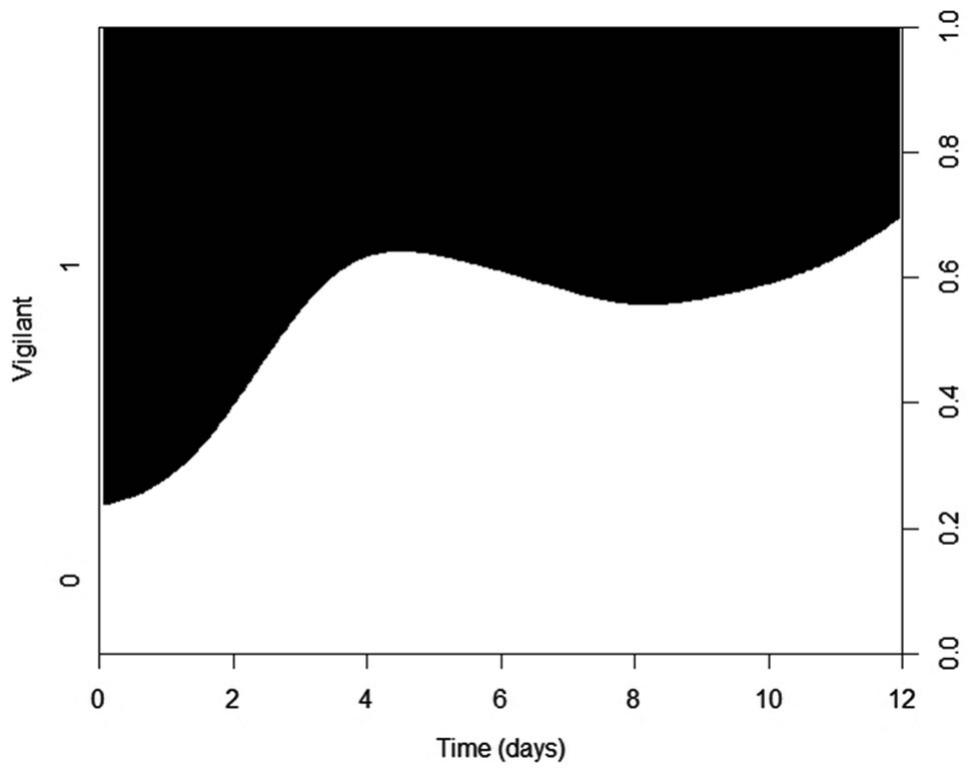
Vigilant behavior (presence or absence) of red foxes (*n* = 73) at sites with lynx scats in relation to time (0–12 days) since the experiment started in the Białowieża Primeval Forest, Poland, during autumn 2012. *Black area* shows the presence (1) of vigilance, and* white area* shows its absence (0). Fox behavior was recorded by movement- and body-heat-triggered passive sensor cameras

During the recorded fox visits used for the behavioral analyses (*n* = 98) only one class of behavior was displayed at control sites [passing by (*n* = 22), vigilance (*n* = 1), sniffing (*n* = 1) or scent marking (*n* = 1)], and one or more different behaviors at scat sites. Sniffing (*n* = 60) was the most common behavior at scat sites, followed by scent marking (*n* = 38), vigilance (*n* = 35), and passing by (*n* = 9, Fig. 1). Vigilant behavior, sniffing and scent marking occurred more often at scat sites compared to control sites (Table 1). In contrast, fox passing by were recorded more often at control sites than at scat sites (Table 1). Vigilance was the only behavior type influenced by time since the experiment started, with foxes being more vigilant at scat sites during the beginning of the 12 consecutive days, i.e., when deployed lynx scats were fresher (Table 2; Fig. 2).

**Table 1:** The effects of treatment (site with added lynx scat or control site^a^) on the presence/absence of different behaviors of red fox (*n* = 98 visits) in the Białowieża Primeval Forest, Poland, during autumn 2012

Behavior	*β*	SE	*p*	Odds ratio^b^	95% CI for odds ratio	
					Lower	Upper
Passing by	−3.954	0.711	<0.001	0.019	0.004	0.068
Vigilant	3.096	1.047	0.003	22.105	4.310	405.522
Sniffing	4.707	1.065	<0.001	110.769	20.601	2070.578
Scent marking	3.260	1.047	0.002	26.057	5.082	478.037

^a^The control site is the reference in the analyses

^b^Odds ratio (e^*β*^) quantified the change in the probability of a behavior being shown relative to the change in the fixed factor

**Table 2:** The effects of time since the experiment started (0–12 days) and red fox over-marking (presence or absence of red fox scent marks^a^) on the presence/absence of different behaviors of red fox (*n* = 73) at experimental sites with lynx scats added in the Białowieża Primeval Forest, Poland, during autumn 2012

Behavior	Factor	*β*	SE	*p*	Odds ratio^b^	95% CI for odds ratio	
						Lower	Upper
Passing by	Time	0.167	0.124	0.181	1.181	0.927	1.527
	Fox scent marks	−1.648	0.861	0.056	0.192	0.030	0.966
Vigilant	Time	−0.162	0.086	0.058	0.850	0.712	1.000
	Fox scent marks	0.209	0.546	0.702	1.232	0.428	3.708
Sniffing	Time	−0.156	0.111	0.161	0.856	0.680	1.060
	Fox scent marks	1.851	0.770	0.016	6.366	1.525	32.890
Scent marking	Time	−0.099	0.088	0.257	0.905	0.756	1.070
	Fox scent marks	1.499	0.581	0.010	4.475	1.496	14.986

^a^The absence of red fox scent marks is the reference in the analyses

^b^Odds ratio (e^*β*^) quantified the change in the probability of a behavior being shown relative to the change in the fixed factor and a one-unit change in the covariate

All over-marking (with urine in all cases) of the lynx scat (*n* = 11, Fig. 1) by fox began with the fox sniffing at, or close to, the lynx scat before the actual marking. Foxes over-marked once (*n* = 5), twice (*n* = 5) or five times (*n* = 1), at each occasion on top or close to their previous marking. Multiple over-marking by foxes (*n* = 27; Fig. 1) occurred at seven scat sites. Multiple over-marking occurred once (*n* = 13), twice (*n* = 13) or three times (*n* = 1) at each occasion (with urine in all cases, except one with both urine and feces). Fox behavior was influenced by foxes previously visiting scat sites. They sniffed and scent marked more frequently and were less likely to just pass by when the lynx scat had been over-marked previously by fox (Table 2).

## Discussion

Foxes visited lynx scat sites more frequently, stayed there longer, and displayed a higher frequency of behaviors related to attraction (sniffing and scent marking, including over-marking and multiple over-marking) than they did at control sites. Fox attraction to the lynx olfactory cue was obvious, and the frequency of vigilant behavior was also higher at scat sites than at control site. This can be interpreted as an indication of fear. Nevertheless, interpreting fox behavior based on the presence-absence of a lynx olfactory cue may be misleading and must be done with caution. Our study lacked a non-predator scat control, which would have been beneficial to elucidate if foxes commonly urinate on any type of scat or if this behavior is unique for lynx scats. However, to our knowledge, our study is the first to show that foxes, contrary to our assumption, express two opposing responses, attraction and fear, towards an olfactory cue of lynx. Showing the attraction of the mesopredator to the apex predator cue is a remarkable finding of this study, regardless of the absence of a control with a neutral smell, particularly considering the fact that mammals are clearly able to identify types of smell donor (e.g., Wikenros et al. 2015). This result is particularly striking bearing in mind that foxes are often killed by lynx as they are likely regarded as prey (Linnell et al. 1998), and therefore would be expected to strongly avoid sites with apparent cues of lynx presence. In contrast, they were attracted to them. The higher frequency of fox vigilant behavior at sites with lynx scats also decreased with the lynx scats’ age. This is evidence that the methodological approach we used to collect and store the scats provided sufficiently fresh material, and suggests that fear intensity may also be dependent on the time passed after the presence of the apex predator.

Foxes may face a trade-off between the risk of interspecific killing (Helldin et al. 2006; Elmhagen et al. 2010) and the potential benefit from food availability in the form of carrion that may be left by an apex predator (Jędrzejewski et al. 1993; Jobin et al. 2000; Selva et al. 2005). While the fox is attracted to the scat to retrieve information from olfactory cues, it still needs to be alert because of the potential risk posed by an apex predator in the vicinity. Fox displaying vigilance when approaching a scat reflect both fear and attraction to the scat. In this context, we interpret the observed vigilance, which was most intense when scats were still fresh, as an antipredator response. The fact that foxes did not avoid sites with lynx scats suggests that the information they gain may be beneficial, i.e., a fox might need to adjust its behavior to decrease its risk of being killed (Apfelbach et al. 2005). Scent marks may not only provide information of the species (Wikenros et al. 2015), but also individual characteristics of the animal, such as sex or different individuals (Johnson 1973; Sokolov et al. 1996; Ferkin 2015). Such information may help a fox estimate the proximity to, and the risk of, encountering lynx (Kats and Dill 1998).

In addition, predator scats may reveal the existence of lynx-killed prey in the vicinity, and olfactory cues can also provide information on the diet of the predator, due to undigested remains (such as hair and bones) in the feces (Mason et al. 1994; Nolte et al. 1994; Kats and Dill 1998; Mirza and Chivers 2003; Apfelbach et al. 2015). Apparent attraction of the foxes to the sites with lynx scats suggests that mesopredators are interested in the olfactory cue of another predator species. During our study we also noted visits at both scat and control sites by three other mesopredator species [marten (*Martes* spp.), badger (*Meles meles*) and raccoon dog (*Nyctereutes procyonoides*)]. Similar to foxes, martens over-marked the lynx scats, expressed vigilant behavior and spent a longer time at them compared to control sites. However, the sample size was too small for statistical analyses and comparison with fox behavior, but the data do suggest that martens did not avoid lynx scat sites either. That mesopredators respond with both attraction and fear contrasts to the type of reactions observed in ungulate prey species, which showed only fear (higher vigilance or avoidance) when they were exposed to scats from apex predators in the same ecosystem (Kuijper et al. 2014; Wikenros et al. 2015). Interestingly, a similar attraction to wolf (*Canis lupus*) scats was observed in wild boar (*Sus scrofa*, Kuijper et al. 2014). However, wild boar did not show any behavioral responses to these (e.g., higher vigilance or avoidance) indicating a perceived predation risk near wolf (Kuijper et al. 2014) or lynx scats (Wikenros et al. 2015), as shown for foxes in this study. Because the wild boar is omnivorous and plays only a secondary role in the wolf diet, and is only occasionally killed by lynx in BPF (Okarma et al. 1997), these findings support the possibility that the smell of the predators’ scats is used as a source of information on the distribution of potential food in the case of animals that are not a main prey species. Taken together, the reactions of foxes in our study may be a combination of antipredatory behavior and a commensal interaction between predatory species.

For many terrestrial mammals scent marks may act as signals that provide information on mates, resources and predation risk (Johnston 1983, 1990; Thiessen and Rice 1976; Roberts 2007), which in turn increases the receiver’s fitness, i.e., survival and reproduction (Apfelbach et al. 2005; Ferkin 2015). A particular behavior observed in foxes was over-marking the lynx scat with urine or feces. The function of scat over-marking in the case of intraguild interspecific relationships is unclear. Over-marking behavior has been hypothesized to play an important role in intraspecific communication regarding competition, mate attraction, mate guarding, or group cohesion (Ferkin and Pierce 2007), but to our knowledge, there are no hypotheses explaining interspecific communication in the literature. It could be that lynx scats are yet another other object, such as rocks, trees, and carrion, acting as substrate for the fox to scent mark in its intraspecific communication. Alternatively, over-marking may record whether the lynx scat has already been investigated (Henry 1977), or it may mask the presence of the underlying scent mark (Johnston et al. 1994; Ferkin and Pierce 2007). Over-marking also seemed to trigger multiple over-marking, as 64% of the scats over-marked by foxes were repeatedly over-marked later on by foxes. Leo et al. (2015) showed that fox in New South Wales, Australia, altered their behavior not only when exposed to dingo (*Canis dingo*) odor, but also when exposed to odor from unfamiliar conspecifics. Interference effects by a conspecific may have a similar effect to that of a relatively rare apex predator in their study ecosystem (Leo et al. 2015). In our study it was not possible to determine if multiple over-marking was done by the same or different individuals.

Our study showed that an olfactory cue of lynx triggered fox behavior that indicated both attraction as well as fear. We suggest that foxes extract useful information from apex predator scat, which may help them to find food sources and to estimate the risk of encountering a larger competitor and eventual predator. The role of over-marking lynx scats by fox, as well as the reasons for multiple over-marking, remain unclear and further research is needed to better understand the role of scent marking in intra- and interspecific interactions.
